# Passive mechanical properties of human gastrocnemius muscle-tendon units

**DOI:** 10.1186/1757-1146-5-S1-I3

**Published:** 2012-04-10

**Authors:** Robert D Herbert, Joanna Diong

**Affiliations:** 1 The George Institute for Global Health, Sydney, NSW 2001, Australia

## Background

The passive mechanical properties of skeletal muscle-tendon units are important because they determine the amount of motion available at joints. Human gastrocnemius muscle-tendon units are of particular interest because this muscle is prone to develop contractures, may have a role in lower limb overuse injuries, and is a common site of muscle tears.

This workshop provides an introduction to what is known of the passive properties of skeletal muscle-tendon units, focussing on human gastrocnemius muscle-tendon units. The workshop will also provide an introduction to the theory and practice of measuring passive mechanical properties of human gastrocnemius muscle-tendon units in vivo.

## Materials and methods

The mechanical properties of muscle-tendon units have been investigated most frequently using in vitro preparations. Testing of elastic properties most often utilises quasi-static protocols. Dynamic protocols have also been used, particularly in studies that seek also to determine viscous properties.

Several methods have been developed for testing the mechanical properties (usually elastic or pseudo-elastic properties) of human muscle-tendon units in vivo. Changes in length of human gastrocnemius muscle-tendon units may be estimated from changes in ankle and knee angles if moment arms are known. Fascicle lengths can be measured with ultrasound imaging or MRI. Recently methods have been developed for measuring sarcomere lengths using invasive and minimally invasive techniques. Achilles tendon force can be measured using invasive methods such as fibre optic transducers. The length-tension properties of the Achilles tendon can be estimated using non-invasive methods during isometric contractions.

This workshop focuses on a method developed by our research team for non-invasive measurement of the passive length-tension properties of human gastrocnemius muscle-tendon units [1], as well as length-tension properties of muscle fascicles and tendons [2]. The method involves measuring ankle stiffness at a range of knee angles.

## Conclusions

A range of methods is available for measuring the mechanical properties of human gastrocnemius muscle-tendon units in vivo.
